# The Correlations between Clinical Features, Dermoscopic and Histopathological Findings, and Treatment Outcomes of Patients with Pitted Keratolysis

**DOI:** 10.1155/2021/3416643

**Published:** 2021-10-25

**Authors:** Penvadee Pattanaprichakul, Kanokvalai Kulthanan, Sumanas Bunyaratavej, Sasima Eimpunth, Thanaporn Rungruang, Pattriya Chanyachailert, Punyawee Ongsri, Poramin Patthamalai, Kanyalak Munprom, Charussri Leeyaphan

**Affiliations:** ^1^Department of Dermatology, Faculty of Medicine Siriraj Hospital, Mahidol University, Bangkok, Thailand; ^2^Department of Anatomy, Faculty of Medicine Siriraj Hospital, Mahidol University, Bangkok, Thailand

## Abstract

**Background:**

Pitted keratolysis (PK) is a superficial bacterial infection diagnosed mainly by clinical manifestations. Current data on its dermoscopic and histopathological findings, and the correlation of those findings, are limited.

**Objectives:**

To evaluate the clinical manifestations, dermoscopic, and histopathological findings of PK and to determine the correlations.

**Methods:**

Forty naval cadets with PK and five cadets with normal feet were enrolled this cohort study and provided informed consent. Dermoscopy was independently applied and evaluated by 2 dermatologists. Shave biopsies were performed on 37 patients with PK.

**Results:**

Pits were the most common dermoscopic finding (88.1%). The dermoscope had more sensitivity for the detection of PK than the naked eye examinations. Apart from the pits and the presence of bacteria, the most common histopathological finding for PK was color alteration of keratin. The presence of bacteria correlated with interrupted dermatoglyphic lines and the color alteration of keratin. Moreover, the presence of bacteria at the base of pits was related to worse treatment outcomes.

**Conclusions:**

Dermoscopy is a useful tool for PK diagnosis. Color alteration of keratin is another histopathological finding for PK. The presence of bacteria is associated with worse treatment outcomes.

## 1. Introduction

Pitted keratolysis (PK) is a superficial bacterial infection caused by many organisms, such as *Corynebacterium* spp., *Actinomyces* spp., *Dermatophilus* spp., and *Kytococcus sedentarius* [1, 2]. Patients usually complain of malodor and excessive sweating and sometimes of a burning sensation [3, 4]. The disease commonly affects males, and it is associated with occupations requiring the wearing of occlusive footwear, such as soldiers, industrial workers, and farmers [3]. Alteration of the skin pH, hyperhidrosis, and prolonged occlusion can precipitate bacteria to proliferate and produce enzymes that degrade the keratin of the stratum corneum [4].

The diagnosis of PK is primarily based on its clinical manifestations [4]. Thus, its dermoscopic and histopathological findings have only been investigated by a few studies. Moreover, the known correlations between the clinical manifestations, dermoscopic findings, and histopathological findings are currently limited. To assess this gap, this study is aimed at evaluating the clinical manifestations, dermoscopic and histopathological findings, and the treatment outcomes of PK patients and identifying the correlations between those factors.

## 2. Materials and Methods

Before commencement of this cohort study, its protocol was approved by the Siriraj Institutional Review Board of the Faculty of Medicine Siriraj Hospital, Mahidol University (Si386/2016). Naval cadets aged 18 years or older who were attending the Naval Rating School of the Royal Thai Navy were enrolled. Informed consent was obtained from all participants before their feet were clinically examined by dermatologists. The clinical manifestations and degree of foot odor were assessed, and dermoscopy was performed on all 45 participants. Shave biopsies were subsequently performed on the 37 patients who were diagnosed with PK via the clinical assessments or the dermoscopic findings. The biopsies were performed on the most severe foot lesion areas.

The dermoscopic findings were examined by 2 independent dermatologists. The characteristics of the findings are illustrated in Supplementary Figure 1. The interobserver agreement for the dermoscopic findings was established by the percentage of observed exact agreements and kappa. As to the histopathology, the specimens were stained with hematoxylin and eosin, Gram stain, and periodic acid–Schiff. With regard to the PK treatments, 4% chlorhexidine scrub and 4% erythromycin gel were randomly prescribed for 4 weeks, in accordance with the protocol of a recent study [5]. The outcomes—degree of foot odor and lesions 2 months after treatment—were analyzed to determine the correlations between the clinical, dermoscopic, and histopathological findings.

The associations between the histopathological findings and the qualitative variables (namely, the clinical manifestations and dermoscopic findings) were assessed using Fisher's exact test, whereas the Mann–Whitney *U* test was used for quantitative variables. Statistical significance was set at 0.05. The statistical data were analyzed with SPSS Statistics for Windows (version 18.0; SPSS Inc., Chicago, Ill., USA).

## 3. Results

Of the 45 cadets enrolled in this study, 40 were clinically diagnosed with PK. The remaining 5 cadets had normal foot appearances. All Thai naval cadets were male, with a mean age of 19.8 (SD, ±1.0) years. Most of the PK cases complained of excessive sweating of the feet (95%), itchy feet (87.5%), feet pain (85%), and foot malodor (82%). More than half of the cadets (62.5%) had previously observed pits at their feet, 55% of cadets had scale on soles, and only 10% of cadets had itchy papules or vesicles on the feet. Moreover, history of precipitating factors including wearing wet socks for long periods of time (91.1%), walking barefoot outdoors (88.9%), wearing combat shoes for longer than 8 hours (86.7%), and walking barefoot indoors (57.8%) was reported.

**Figure 1:**
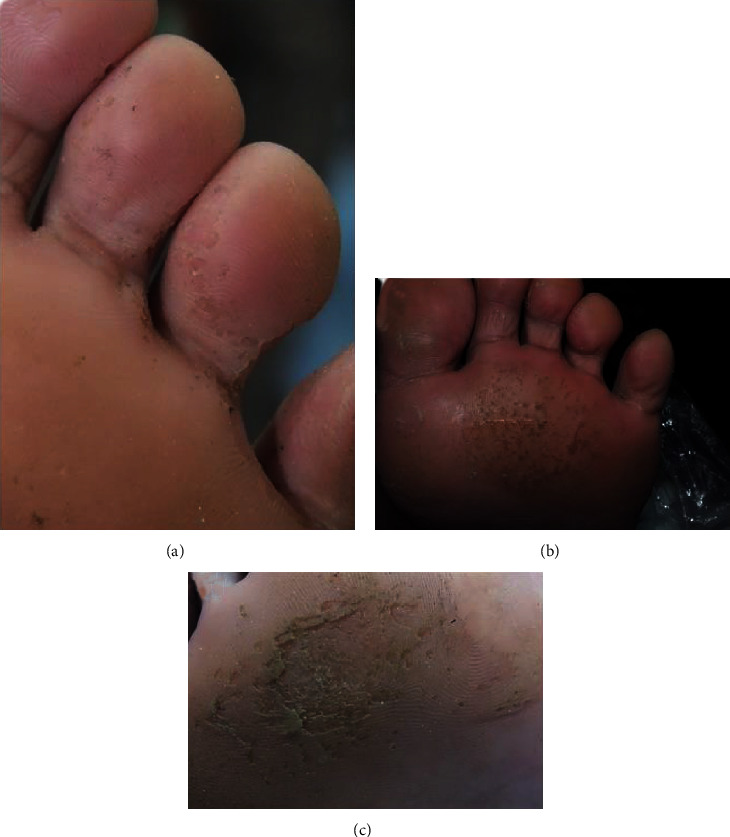
Representative clinical images of pitted keratolysis at toes (a) and forefeet (b and c).

The dermoscopic findings were independently assessed by 2 dermatologists, and there was strong observer agreement (97.8%; *κ*, 0.928). Two cadets with normal foot appearances by clinical examination were diagnosed with PK by dermoscopy. In all, 42 cadets with PK were included in the analysis. The mean visual analog scale score for their foot odor was 4 (SD, ±1.8), and the median Dermatology Life Quality Index score was 8. The common PK location was the forefoot, and the mean area of pit involvement was 40% of the whole-foot surface area. However, the area of involvement was not correlated with the severity of foot odor or the Dermatology Life Quality Index score. The dermoscopic findings of all 42 cadets with PK are listed in Table 1. Pits of various sizes and configurations were detected in 37 (88%) patients by dermoscope. A scattered arrangement and interrupted dermatoglyphic lines of the pits were observed in 86.5% and 89.2% of the patients, respectively. There was no association between the dermoscopic findings and foot odor severity. The representative clinical images of PK were shown in Figure 1.

**Figure 2:**
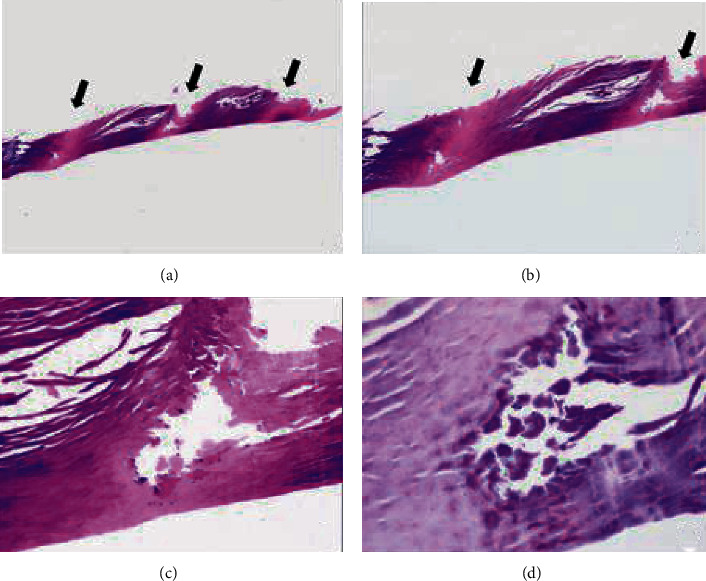
The histopathology demonstrated color alteration of the keratin with alternate light and dark columns (a, b). Alternate dark and light vertical bands with depressed area corresponding to the light columns (arrow) (Hematoxylin and Eosin ×100 and ×200, respectively, a, b). Depressed area with empty space represented the pit with cluster of bacteria along the side and at the bottom of the pit ((c) Hematoxylin and Eosin×400 and (d) Periodic-acid Schiff ×600).

**Figure 3:**
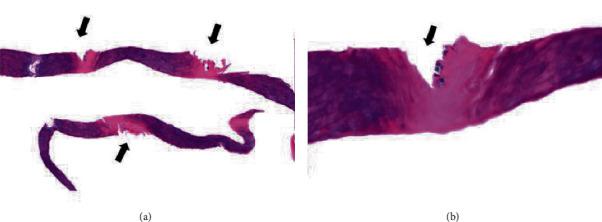
The histopathology showed columns of alternate light and dark color of the keratin with pitting area corresponding to the light columns (arrow) ((a) Hematoxylin and Eosin ×100). Clusters of bacteria are present along the side of the pit ((b) Hematoxylin and Eosin ×400).

In order to conduct histopathological evaluations, shave biopsies were performed on 37 of the 42 patients who had been diagnosed with PK by clinical or dermoscopic examination. The most common histopathological findings were pits related to defects in the stratum corneum (91.9%), color alteration of keratin (73%) (Figure 2, Figure 3), and parakeratosis (13.5%). Parakeratosis was observed in 5 cases and associated with white opaques in the dermoscopic findings (*P* = 0.037). Bacteria—detected in 31 (83%) patients—were only found in 2 places: the walls of pits (67.7%) and both the walls and the bases of the pits (32.3%).

The clinical findings, dermoscopic features, and histopathology were analyzed to identify any associations. While the presence of bacteria in the histology tended to relate to a worse foot odor severity and a worse Dermatology Life Quality Index score, there was no statistical significance. Moreover, the presence of bacteria was related to lost or interrupted dermatoglyphic lines in the dermoscopic findings, and a color alteration of keratin in the histopathology (Table 2).

Treatment (either a 4% chlorhexidine scrub or a 4% erythromycin gel) was prescribed for 27 of the cadets with PK. An analysis was made of the treatment outcomes (degrees of foot odor and foot lesion improvement) as well as the dermoscopic and histopathological findings (Table 3). The presence of bacteria at the wall and bases of the pits were found to be significantly related to worse treatment outcomes.

## 4. Discussion

PK is a common health problem and strongly impacts patients' quality of life, especially in tropical and humid countries [4–6]. The diagnosis is based on clinical findings [4]. Nowadays, a dermoscope is a noninvasive and helpful diagnostic tool which is used for many dermatologic diseases. The most common dermoscopic finding of PK is an abundance of pits [7]. A wide range of pit characteristics can be revealed by dermoscopy, and this study presented detailed dermoscopic findings for PK. Other than pits, white and black opaques were observed. The white opaques were correlated with parakeratosis in the histology and scales in the clinical examinations. This study also confirmed that dermoscopy is more sensitive than clinical examinations by the naked eye in terms of the diagnosis of PK. Thus, a dermoscope can facilitate the visualization of pits in patients who have foot malodor with an otherwise grossly normal foot appearance. However, the chief limitation of a dermoscope is its small visual field, which means that physicians need to apply the dermoscope to the entire foot area.

The histopathology of PK mainly revealed pits and the presence of bacteria [3, 4, 8–10]. The pits corresponded to stratum corneum defects, and the bacteria were detected at the base or wall of the pits. Color alteration of keratin was another important histological finding, being found in 73% of the study cohort. These findings correlate with the presence of bacteria reported by Zaias et al., whose investigation revealed color alterations of keratin in histopathology and darkened depressions corresponding to microcolonies of bacteria [9]. In contrast to our study, the light columns of keratin were found microcolonies of bacteria. Recently, in vivo reflectance confocal microscopy (RCM), a noninvasive technique to diagnose various skin diseases, was used to provide a horizontal “point of view” of the skin. Previous studies reported the correlations between dermoscopy, RCM, and histopathology in several skin diseases [11, 12]. Since shave biopsy was conducted in this study, thin parts of tissues were obtained. Therefore, only traditional vertical histopathology sections were evaluated. Further studies are needed to demonstrate the association between dermoscopy, RCM, and histopathology in pitted keratolysis.

The presence of bacteria, found in 74% of the PK patients in this study, was the key feature of PK disease severity and the treatment outcomes. The greater the severity of foot malodor was, the worse was the quality of life of the patients with bacteria. Moreover, interrupted dermatoglyphic lines—which imply the presence of large and continuous pit extensions—were related with the presence of bacteria. Thus, the presence of bacteria in the histopathology may imply a greater severity of PK. Furthermore, the PK treatment of patients whose bacteria were found at the base of pits proved to be more difficult than that of patients without bacteria or with bacteria only on pit walls. Thus, prolonged treatment and close follow-up are likely to be needed for this group.

This study had some limitations. Firstly, the sample size was small, and the participants were restricted to male cadets of a similar age group. Moreover, although the dermoscopic and histological examinations of each participant were performed in the area with the worst PK severity as determined by naked eye examination, that area may not actually be representative of the whole affected area. Lastly, bacterial culture and RCM which provides the horizontal “point of view” of the skin were not performed.

## 5. Conclusions

A dermoscope can facilitate the visualization of pits and help clinicians to diagnosis PK in patients with foot malodor. Apart from the presence of pits in histology, the alteration of keratin color is an important histological indicator of PK. Moreover, the presence of bacteria at the base of pits is associated with worse treatment outcomes.

## Figures and Tables

**Table 1:** Associations between pitted keratolysis (PK) diagnoses and dermoscopic findings of 45 cadets.

Dermoscopic findings	Number (%)		*P*value
	PK (*n* = 42)	Non-PK (*n* = 3)	
Pit	37 (88.1)	0	0.004^∗^
Pit size			
Same	19/37 (51.4)	—	
Mixed	18/37 (48.6)	—	
Pit configuration			
Same	12/37 (32.4)	—	
Mixed	25/37 (67.6)	—	
Pit arrangement			
Clustered	5/37 (13.5)	—	
Scattered	32/37 (86.5)	—	
Pit characteristics			
Geometric	3/37 (8.1)	—	
Interrupted dermatoglyphic lines	33/37 (89.2)	—	
Bead sign	8/37 (21.6)	—	
White opaque	10 (23.8)	2 (66.7)	0.169
Black or brown opaque	3 (7.1)	0	1.000

^∗^ *P* < 0.05 was statistically significant.

**Table 2:** Associations between clinical manifestations and presence of bacteria found in histopathology.

Clinical manifestations	Number (%)		*P* value
	No bacteria presence (*n* = 6)	Bacteria presence (*n* = 31)	
Clinical severity			
Involvement area (% of total foot area)	50.8	51.7	0.804
VAS of foot odor (mean ± SD)	2.8 ± 1.9	4.0 ± 2.0	0.175
DLQI: median (P25, P75)	6.0 (2.8, 7.3)	8.0 (3.0, 15.0)	0.432
Dermoscopic findings			
Pit	6 (100.0)	26 (83.9)	0.567
Pit size			
Same	3/6 (50.0)	11/26 (42.3)	1.000
Mixed	3/6 (50.0)	15/26 (57.7)	
Pit configuration			
Same	1/6 (16.7)	7/26 (26.9)	1.000
Mixed	5/6 (83.3)	9/26 (73.1)	
Pit arrangement			
Clustered	1/6 (16.7)	3/26 (11.5)	1.000
Scattered	5/6 (83.3)	23/26 (88.5)	
Pit characteristics			
Geometric	1/6 (16.7)	2/26 (7.7)	0.476
Interrupted dermatoglyphic lines	3/6 (50.0)	25/26 (96.2)	0.015∗
Bead sign	2/6 (33.3)	6/26 (23.1)	0.625
White opaque	0	7 (22.6)	0.571
Black or brown opaque	0	3 (9.7)	1.000
Histopathological findings			
Pits	4 (66.7)	30 (96.8)	0.062
Parakeratosis	0	5 (16.1)	0.567
Color alteration of keratin	2 (33.3)	25 (80.6)	0.035^∗^

^∗^ *P* < 0.05 was statistically significant. Abbreviations: DLQI: Dermatology Life Quality Index; VAS: visual analog scale.

**Table 3:** Associations between dermoscopic-histopathological findings and improvement of foot odor and lesions after 2 months of treatment.

	Foot odor			Foot lesions		
	No improvement (*n* = 6)	Improvement (*n* = 21)	*P* value	No improvement (*n* = 8)	Improve ment (*n* = 19)	*P* value
Clinical findings at baseline						
Severity of foot odor (VAS)ǂ	4.8 ± 1.5	3.9 ± 1.9	0.29	5.2 ± 1.0	3.6 ± 1.8	0.04^∗^
Mean quality-of-life scoreǂ	13.5 ± 7.4	6.7 ± 6.9	0.04^∗^	16.4 ± 7.1	4.7 ± 4.3	0.05^∗^
Involvement area (% of total foot area)	41.7	53.3	0.26	40.5	55.7	0.12
Dermoscopic findings						
Pit	4 (66.7)	19 (90.5)	0.15	6 (75.0)	17 (89.5)	0.56
Size						
Same	1/4 (25.0)	10/19 (52.6)	0.32	2/6 (33.3)	9/17 (52.9)	0.64
Mixed	3/4 (25.0)	9/19 (47.4)		4/6 (66.7)	8/17 (47.1)	
Configuration						
Same	0	7/19 (36.8)	0.27	1/6 (16.7)	6/17 (35.3)	0.62
Mixed	4/4 (100.0)	12/19 (63.2)		5/6 (83.3)	11/17 (64.7)	
Arrangement						
Clustered	0	3/19 (15.8)	1.00	1/6 (16.7)	2/17 (11.8)	1.00
Scattered	4/4 (100.0)	16/19 (84.2)		5/6 (83.3)	15/17 (88.2)	
Characteristics						
Geometric	2/4 (50.0)	1/19 (5.3)	0.07	1/6 (16.7)	2/17 (11.8)	1.00
Interrupted dermatoglyphic lines	4/4 (100.0)	16/19 (84.2)	1.00	6/6 (100)	14/17 (82.4)	0.54
Bead sign	0	5/19 (26.3)	1.00	2/6 (33.3)	3/17 (17.6)	0.58
White opaque	1 (16.7)	4 (19.0)	1.00	1 (12.5)	4 (21.1)	1.00
Black/brown opaque	1 (16.7)	1 (4.8)	0.40	2 (25)	0	0.02^∗^
Histopathological findings						
Pits	6 (100.0)	20 (95.2)	1.00	8 (100.0)	18 (94.7)	1.00
Parakeratosis	2 (33.3)	2 (9.5)	0.21	2 (25.0)	2 (10.5)	0.56
Color alteration of keratin	6 (100.0)	16 (76.2)	0.56	8 (100.0)	14 (73.7)	0.11
Presence of bacteria	5 (83.3)	19 (90.5)	0.55	8 (100.0)	16 (84.2)	0.53
Location of bacteria						
Only at the walls of pits	1/5 (20.0)	14/19 (73.7)	0.05^∗^	3/8 (37.5)	12/16 (75.0)	0.04^∗^
At the walls and bases of pits	4/5 (80.0)	5/19 (26.3)		5/8 (62.5)	4/16 (25.0)	

^∗^ *P* < 0.05 was statistically significant. ǂ The statistics are presented as mean ± SD. Abbreviation: VAS: visual analogue score.

## Data Availability

The data used to support the findings of this study are included within the article.

## Supplementary Materials

Supplementary Materials Supplementary Figure 1: the characteristics of each dermoscopic finding.
