# Supplementary material for: The Correlations between Clinical Features, Dermoscopic and Histopathological Findings, and Treatment Outcomes of Patients with Pitted Keratolysis
Source: Biomed Res Int. 2021 Oct 25;2021:3416643. doi: 10.1155/2021/3416643 (PMC8560252; doi:10.1155/2021/3416643)
Supplement: Supplementary Materials — Supplementary Figure 1: the characteristics of each dermoscopic finding. [file 3416643.f1.pdf]

**Supplementary figure 1.** The characteristics of each dermoscopic finding

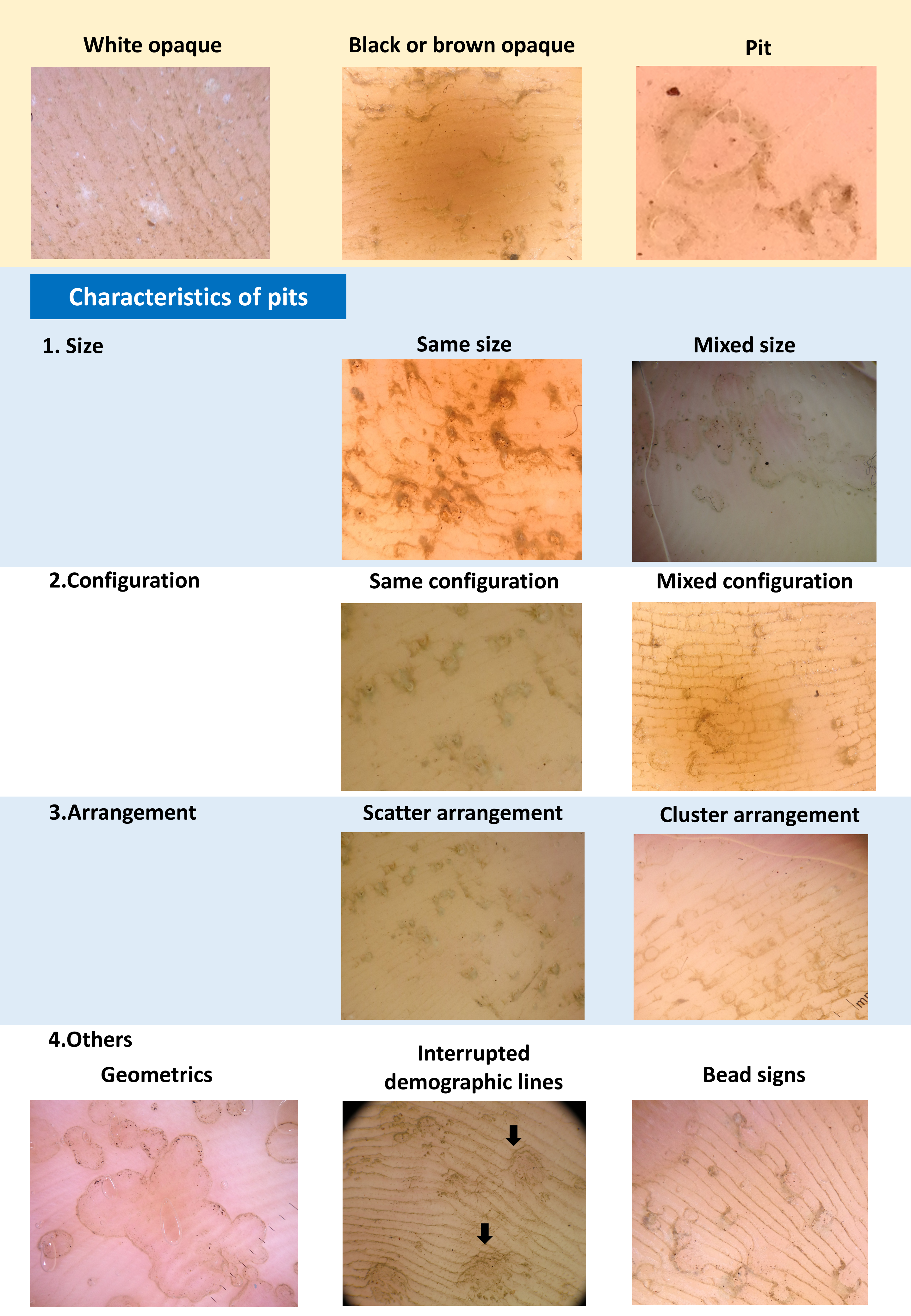
White opaque
Black or brown opaque
Pit
Characteristics of pits
1. Size
Same size
Mixed size
2.Configuration
Same configuration
Mixed configuration
3.Arrangement
Scatter arrangement
Cluster arrangement
4.Others
Geometrics
Interrupted demographic lines
Bead signs
